# Transhumanism: Integrating Cochlear Implants With Artificial Intelligence and the Brain-Machine Interface

**DOI:** 10.7759/cureus.50378

**Published:** 2023-12-12

**Authors:** Aynur Aliyeva

**Affiliations:** 1 Otolaryngology - Head and Neck Surgery, The Catholic University of Korea, Seoul St.Mary Hospital, Seoul, KOR

**Keywords:** hearing rehabilitation, auditory rehabilitation, artificial intelligence (ai), brain-machine interfaces (bmi), cochlear implants (ci), transhumanism

## Abstract

The integration of cochlear implants (CI) with brain-machine interfaces (BMIs) and artificial intelligence (AI) within the framework of transhumanism is revolutionary and this editorial highlights how this synergy can transcend human sensory experiences and auditory rehabilitation. The potential of this amalgamation extends beyond restoring auditory function to enhancing human capabilities, marking a transformative step towards a future where technology harmoniously extends human faculties.

## Editorial

In an era fervently fueled by rapid technological advancements, the transhumanist approach stands at the forefront, encouraging a symbiotic relationship between humans and technology. Central to this conversation is the revolutionary prospect of integrating cochlear implantation (CI) with brain-machine interfaces (BMIs) and artificial intelligence (AI), charting an unprecedented course in augmenting human sensory experiences.

Transhumanism is more than a philosophical movement; it is an exploratory field resonating between science and philosophy. It proposes a future where we transcend beyond our biological confines, leveraging technology to enhance human potential, thereby ushering in a transformative phase in human evolution. CIs are remarkable medical devices that restore auditory perceptions by directly stimulating the auditory nerve, bypassing impaired cochlear functionalities. Parallelly, BMIs facilitate a direct communication pathway between the brain and external devices. This is not just a connection but a rich dialogue where machines can interpret and respond to neural signals, paving the way for harmonized man-machine interactions [1].

Looking at CIs through a BMI lens reveals a landscape where the auditory processes become finely tuned with the mechanistic precision of a machine. Crowson et al.'s study highlighted that CIs function as specialized BMIs, enhancing our understanding of the nexus between neural networks and computational technologies [2].

Venturing further, the marriage between CIs and AI heralds transformative potentials. Waltzman and Kelsall delve deep into this, illustrating the prospects of AI-driven programming of cochlear implants, a venture that promises personalized auditory experiences fine-tuned to the individual's neural landscape [3].

Leveraging AI in CIs embodies a transformative step for hearing rehabilitation. For instance, the computer-assisted FOX (Fitting to Outcomes eXpert) tool (Otoconsult BV, Antwerp, Belgium) as elaborated by Wathour et al., signifies a promising stride, showcasing enhanced outcomes in the implant fitting processes [4]. This convergence heralds a future where the hearing-impaired regain auditory functionalities and experience an enriched auditory perception, closing the gap between disability and ability.

The future is replete with opportunities and potential. Zhang et al. outline the myriad of possibilities and challenges that await us at the confluence of BMIs and AI [5]. The transhumanist trajectory leans towards a harmonious integration of technology and biology, foreseeing a world where augmentations are not just for restorative purposes but for transcending human capabilities.

In conclusion, the amalgamation of CIs with AI and BMIs, steered by transhumanist philosophies, not only champions a novel pathway in auditory rehabilitation but beckons a future where the human sensory experience is dramatically enhanced, potentially reaching new heights previously deemed unattainable. It heralds a transformation where technology becomes a harmonious extension of human faculties, navigating a course toward a future rich with possibilities, a testament to human innovation and the relentless pursuit of progress.
